# Count data regression modeling: an application to spontaneous abortion

**DOI:** 10.1186/s12978-020-00955-2

**Published:** 2020-07-08

**Authors:** Prashant Verma, Prafulla Kumar Swain, Kaushalendra Kumar Singh, Mukti Khetan

**Affiliations:** 1 Department of Statistics, Banaras Hindu University, Varanasi, India; 2 Analytics Department, Global IT Center, SBI, Navi Mumbai, India; 3 Department of Statistics, Utkal University, Bhubaneswar, Vanivihar India; 4 Department of Mathematics, Indian Institute of Technology (IIT) Bombay, Mumbai, India

**Keywords:** Count data; spontaneous abortion; Poisson model, Negative binomial model, Zero hurdle negative binomial, Zero-inflated negative binomial, Regression

## Abstract

**Background:**

In India, around 20,000 women die every year due to abortion-related complications. In count data modeling, there is sometimes a prevalence of zero counts. This article is concerned with the estimation of various count regression models to predict the average number of spontaneous abortions among women in Punjab and few northern states in India. The study also assesses the factors associated with the number of spontaneous abortions.

**Methods:**

This study includes 27,173 married women of Punjab obtained from the DLHS-4 survey (2012–13) to train the count models. The study predicts the average number of spontaneous abortions using various count regression models, and also identifies the determinants affecting the spontaneous abortions. Further, the best model is validated with other northern states of India using the latest data (NFHS-4, 2015–16).

**Results:**

Statistical comparisons among four estimation methods reveals that the ZINB model provides the best prediction for the number of spontaneous abortions. The study suggests total children born to a woman, antenatal care (ANC) place, place of residence, woman’s education, and economic status are the most significant factors affecting the instance of spontaneous abortion.

**Conclusions:**

This article offers a practical demonstration of techniques designed to handle count outcome variables. The statistical comparisons among four estimation models revealed that the ZINB model provides the best prediction for the number of spontaneous abortions, and it suggests policymakers to use this model to predict the number of spontaneous abortions. The study recommends promoting higher education among women in Punjab and other northern states of India. It also suggests that women must receive institutional antenatal care and have a limited number of children.

## Plain English summary

In India, 10% of total pregnancies end up with abortion, miscarriage, or stillbirth (NFHS-4, 2015–16), and approximately 20,000 women die every year due to abortion-related complications. Northern states of India contribute significantly high to the total number of spontaneous abortions occurring in India.

This article is concerned with the estimation of various count regression models to predict the average number of spontaneous abortion (SA) among women in the state of Punjab in India. Further, the best count regression model is validated with a few northern states of India.

The initial model building process utilizes the data of 27,173 married women of Punjab obtained from the DLHS-4 survey. Various count regression models have been tried and tested to predict the number of SA in Punjab. ZINB model is the best model to predict the scenario, and the same has been validated using DHS-4 (2015–16) data pertaining to few other northern states of India, such as Delhi, Uttar Pradesh, and Haryana.

Woman’s education, antenatal care (ANC) place, total children born to a woman, place of residence, and economic status are found to be the most significant factors affecting the occurrence of spontaneous abortions and the number of SA. The study recommends promoting higher education among women in Punjab and other northern states of India. It also suggests that women must receive institutional antenatal care and limit their family size.

## Background

Abortion is usually misunderstood outside the medical community. In medical science, abortion refers to a loss of a fetus, for any reason, before it can survive outside the womb [1]. The term abortion covers spontaneous termination or miscarriage, as well as a deliberate termination of pregnancy. The term “miscarriage” is often used as a synonym with patients because the word “abortion” is generally associated with the selective termination of pregnancy.

Spontaneous abortion, or miscarriage, is defined as a clinically recognized loss of a pregnancy before 20 weeks of gestation [2, 3]. The World Health Organization (WHO) defines it as expulsion or extraction of an embryo or fetus weighing 500 g or less. 15 to 20% of all clinically recognized pregnancies end up in SA, whereas the total pregnancy loss is estimated to be 30 to 50% of all conceptions [4**–**7]. Vaginal bleeding – light spotting or heavy bleeding with clots – is the most common symptom of spontaneous pregnancy loss. Women usually experience cramps in the lower abdominal portion after the bleeding [8].

Fetal chromosome abnormalities and mutant genes are the most common cause of SA, with chromosome imbalance causing at least 50% pregnancy losses in the first trimester and 20% in the second [9]. SA may also occur as a result of environmental toxins (lead, drugs, and ionizing radiation), infectious agents (viruses and bacteria), uterine abnormalities (malformations, fibroids, cervical insufficiency, post-operative changes), and other maternal or paternal factors (chronic disease) [10]. However, it is challenging to observe these abnormalities without an intensive clinical diagnosis. Besides the above medical causes, there exist several socio-economic and personal factors that cause a pregnancy to end up in a spontaneous loss.

In 2014 [11] suggested that around 20,000 women die every year due to abortion-related complications in India; this makes the theme of this study of considerable policy importance. It is hard to get information regarding induced abortion in India given the legal constraints and the status of education; however, some large scale surveys do provide data on induced and spontaneous abortion to an extent. In India, 4.7% of pregnancies end up as SA, with the percentage being somewhat high (5.9%) in the state of Punjab (Report, DLHS-3), while it has come down to 3.9% in 2012 (DLHS-4).

Looking at the recent (Last 5 years) trend in India, 10% of total pregnancies end up with abortion, miscarriage, or stillbirth (NFHS-4, 2015–16), while the proportion for SA is 6%. The SA is particularly high (10.1%) for women age 15–19 years compared to other age groups; this indicates that miscarriages are significantly high among young women, which shows the recent behavior of spontaneous abortion in India. Almost every northern state shows a high proportion of SA, e.g., Delhi (10.5%), Uttar Pradesh (8.6%), Haryana (6.6%), and Punjab (6.1%). Therefore, these states have been assessed to have a comparative study on spontaneous abortion between Punjab and a few other northern states in India.

At the time of initial model building, NFHS-4 data were not available; therefore, the initial models are trained on DLHS-4 data of Punjab. Further, the best model is validated with a few other northern states data (NFHS-4), which show the high spontaneous abortion rate.

Using the Poisson regression (PR), Negative Binomial (NB), Zero Hurdle Negative Binomial (ZHNB), and Zero-Inflated Negative Binomial (ZINB) models, the present study tries to discover the prevalence of SA in Punjab using the data on women at risk of SA. It also investigates the reasons behind SA in Punjab. An initial version of the abstract has been published in the abstract book of *“The 15th Congress of the European Society of Contraception and Reproductive Health-2018”* [12].

Several studies in the literature support the use of the models mentioned above for count regression [13] used PR and NB regression models to predict the average number of children ever born to women in the U.S. **[**14**]** offered a practical demonstration of regression models recommended for count outcomes using longitudinal predictors of children’s medically attended injuries. Similarly, **[**15**]** made an attempt to model fertility behavior using various count regression models based on religious, educational, economic, and occupational characteristics.

## Methods

This study utilizes the data of the fourth round of the District Level Household and Facility Survey (DLHS-4) 2012–13, undertaken by the Ministry of Health and Family Welfare, Government of India. The DLHS data have been useful in setting benchmarks and assessing the progress of the country after the implementation of the Reproductive and Child Health (RCH) programme. The present study involves 27,173 married women in Punjab who have experienced pregnancy at least once in their reproductive span since only these women are at the risk of miscarriages. We have taken only married women since, In India, due to social constraints, pregnancy before marriage is often considered unethical and not reported usually; this data gap makes us unable to predict the SA before marriage among Indian women.

### Statistical models

The number of SA, considered to be a discrete variable, is a count outcome within the population; hence the Poisson Regression model is an appropriate model to predict the prevalence of SA. We have explored a number of statistical models (PR, NB, ZHNB, and ZINB) to test the ability of each model to determine the factors associated with the prevalence of SA.

#### Poisson model

Let denote the number of SA for the ***i***^*th*^ women. Since the data are in terms of counts, we assume that *Y*_*i*_ follows a Poisson distribution with mean *λ*_*i*_ (mean number of SA). Hence, the probability of observing any specific count *Y*_*i*_ is given in eq. 1:

1 $$ P\left({Y}_i={y}_i\right)=\frac{e^{-{\lambda}_i}{\lambda}_i^{y_i}}{y_i!}\kern4.919997em {y}_i=0,1,2,\dots, \kern3em {\lambda}_i>0 $$

For the further estimation of *λ*_*i*_ please refer to **[**16**].**

#### Negative binomial model

The Poisson regression model is not well-suited for overdispersed data. For such cases, the Negative Binomial model is the best alternative to predict the count variable.

Let *Y*_*i*_ be the number of SA for the *i*^*th*^ women, having a Negative Binomial distribution with parameters r and p (probability of having SA). The probability of observing any specific count of SA *Y*_*i*_ is, as such, given in eq. 2, and the estimation process of the model refers to **[**17**].**

2 $$ P\left(Y={y}_i\right)=\frac{\Gamma \left(y{}_i+r\right)}{y_i!\Gamma r}{p}^r{\left(1-p\right)}^{y_i}\kern2.639999em {y}_i=0,1,2,3,\dots; \kern0.72em 0\le p\le 1\kern0.37em $$

**[**18**]** suggested using ZHNB/ZINB models, where the over-dispersion is due to excess zeros because it underestimates the probability of zeros.

In count data regression modeling, the zeros can be of two types

- Structural/true zeros: In structural zero, a subject is at no risk or very low risk of the event of interest, and the reported outcome variable is zero.
- Sampling/false zeros: In sampling zero, a subject is at high risk of the event of interest, and the reported outcome variable is zero.

It has been noticed that zero hurdle models are more suitable in case of an abundance of structural zeros. In contrast, zero-inflated models should be considered where both types of zeros are present **[**19**]**.

#### Zero hurdle models

The zero hurdle models are mainly used when the excess zeros arise from a population where each individual has equal risk of the event. In the context of the present study, this implies that every woman has equal risk of SA. In this model, the zeros are explained through logistic regression and the positive counts through a zero-truncated negative binomial model. The ZHNB model can be expressed as eq. 3**[**18**]:**

3 $$ f\left({y}_i/{x}_i\right)=\left\{\begin{array}{l}{p}_i\kern26.25em ,\kern0.5em {y}_i=0\\ {}\left(1-{p}_i\right)\frac{\Gamma \left({y}_i+{\alpha}^{-1}\right)}{\left(1-{\left(\frac{\alpha^{-1}}{\alpha^{-1}+{\lambda}_i}\right)}^{1/\alpha}\right)\Gamma \left({y}_i+1\right)\Gamma \left({\alpha}^{-1}\right)}{\left(\frac{\alpha^{-1}}{\alpha^{-1}+{\lambda}_i}\right)}^{1/\alpha }{\left(\frac{\lambda_i}{\alpha^{-1}+{\lambda}_i}\right)}^{y_i},\kern0.5em {y}_i\ge 1\end{array}\right. $$

The ZHNB model produces two sets of results; thus, the hurdle models are also referred to as two-part models. The hurdle model identifies factors that are associated with the presence/absence of SA. On the other hand, the modeling count process furnishes factors that are associated with an increase in the number of SA given that all the women are at an equal risk of SA. It is worthwhile to note that the ZHNB model accounts for over-dispersion only due to excess zeros and not to unobserved heterogeneity in SA among women. Here, unobserved heterogeneity refers to fetal chromosome abnormalities, mutant genes, and other biological factors.

#### Zero-inflated models

Zero-Inflated models are mostly utilized when the excess zeros are a combination of true zeros (structural zeros) and false zeros (sampling zeros). If we consider the zero SA in our target population to be a combination of those with a very low risk of SA (true zeros) and those with a high risk of SA (false zeros), we can use the zero-inflated model to capture not only the over-dispersion due to excess zeros but also unobserved heterogeneity among Indian women.

Considering the mechanism built within the zero-inflated models, true/structural zeros are described through logistic regression, whereas false/sampling zeros through the zero-inflated count model. In the present study, false zero SA implies a woman who is at a “high-risk” of SA but does not have a SA due to chance, excellent medical assistance or care, or because she did not report her abortion history during the survey. Similar to the hurdle model, a zero-inflated model also produces two sets of results. However, the interpretation of the coefficients under the two models is quite different.

Considering the occurrence of structural zero SA with probability *p*_*i*_ under a logistic model and that of zero SA (including at “high risk”/false zero SA) with probability *(1-p*_*i*_*)* under the NB model, having a mean number of SA (λ_i_), the ZINB model can be expressed as eq. 4**[**20**]:**

4 $$ p\left({\mathrm{y}}_i/{x}_i\right)=\left\{\begin{array}{l}{p}_i+\left(1-{p}_i\right){\left(1+\frac{\lambda_i}{\tau}\right)}^{-\tau}\kern8.75em ,{\mathrm{y}}_i=0\\ {}\left(1-{p}_i\right)\frac{\Gamma \left({y}_i+\tau \right)}{y_i!\Gamma \left(\tau \right)}{\left(1+\frac{\lambda_i}{\tau}\right)}^{-\tau }{\left(1+\frac{\tau }{\lambda_i}\right)}^{-{y}_i}\kern2.25em ,{\mathrm{y}}_i=1,2,\dots \end{array}\right. $$

All the models mentioned above have been employed to the relevant data using pscl package in ‘R’ software (Cameron and Trivedi, 1998).

### Model comparisons

The independent features, found to be significant in any of the regression models mentioned above, have been included in all the regression models to maintain the comparative findings in multivariate analysis.

**Fig. 1:**
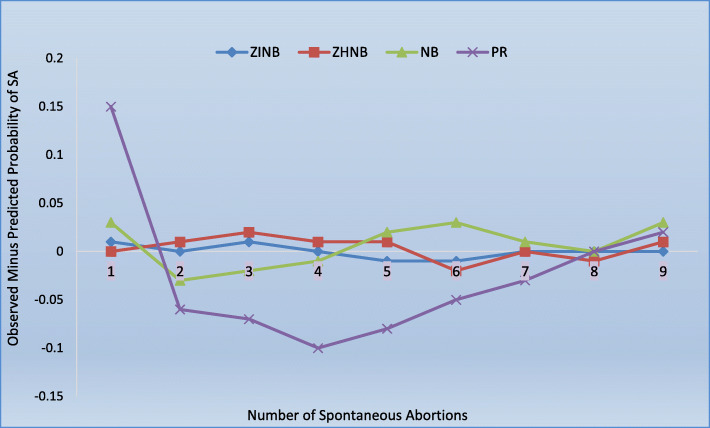
Comparison of Prediction Errors among Various Models

**Fig. 2:**
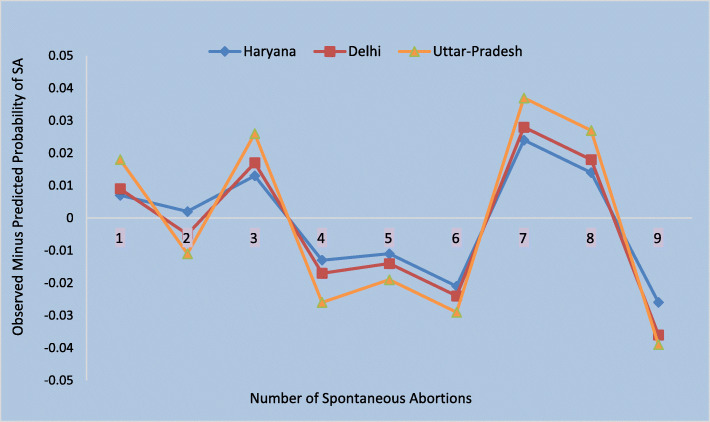
Comparison of Prediction Errors for ZINB among the Various States

For comparing the predictive performance of the used models, various model parameters such as Akaike Information Criterion (AIC), Mean squared prediction error (MSPE), Mean absolute prediction error (MAPE), and log-likelihood has also been obtained. The values of all the above indices used to compare the model fit have been mentioned in Table 1*.* Besides the above criteria, a probability plot (observed probability minus the predicted probability of SA versus the number of SA) is constructed for each model (Fig. 1). The best found model (ZINB) is validated for the other northern states (Delhi, Haryana, Uttar Pradesh) of India, using the latest survey data (NFHS-4, 2015–16). Figure 2 exhibits the accuracies of the ZINB model to predict the SA among the states mentioned above.

**Table 1:** Comparison of model fit characteristics

	Poisson Model	Negative Binomial Model	Zero Hurdle NB Model	Zero Inflated NB Model
**Log Likelihood**	14,405.5	−13,523.5	− 13,460	−13,440
**AIC**	28,826	27,062	26,951	26,911
**MSPE**	10,804	818.81	274.94	255.99
**MAPE**	96.42	32.06	24.95	24.45

## Results

A total of 27,173 married women were found eligible for this study. Of them, 3661 women had experienced termination of pregnancy in the form of SA. The average age of women at marriage is found to be approximately 20 years, and the average number of children ever born to a woman is found to be 2.2. Table 2 exhibits the percentage distribution of the regressors involved in the study.

**Table 2:** Percentage distribution of variables under study

Variables	Percentage Distribution
**Place of Residence**	
Rural	60.70
Urban	39.30
**Caste**	
SC/ST	43.10
O.B.C	13.30
General	43.60
**Wealth Index**	
Low	8.70
Middle	78.15
High	4.45
**Education**	
illiterate	27.66
Primary	30.63
Secondary	31.62
Higher	10.08
**Media Exposure**	
Exposed to media	93.80
Not exposed to media	6.20
**ANC Place**	
Goverment	44.56
Private	49.11
Others	6.34
**Religion**	
Sikh	62.23
Others	37.77

Mean age at marriage = 20.2 years, Average number of total children ever born = 2.22

The model coefficiants support the descriptive statistics and exhibit that the co-factors Antenatal care (ANC) place and women’s age at marriage has been associated with the number of SA across all the models consistently. An excellent harmony could also be observed between the ZHNB and ZINB models in supplying factors associated with the prevalence of SA. In other words, woman’s education, ANC place, place of residence, total children born to a woman, and wealth index are associated with a higher number of SA in both the models. However, only the ZINB model suggests that religion is also associated with a higher number of SA in the high-risk population.

Along with other characteristics of the model, the Pearson chi-square goodness of fit test (*p* < 0.001) indicates that the PR model shows a poor fit for the SA data. In the NB model, the estimated dispersion parameter (α) was 1.12 at a 95% level of significance. This indicates that the over-dispersion exists in the distribution of the target variable. The data suggest that there is a massive number of women who had zero SA, implying an excess of zero SA; this recommends that over-dispersion is due to excess zeros in the number of SA data. All the zeros are considered to arise from the “at same risk” group, justifying the use of the ZHNB model. To estimate false zeros, it is believed that some of these zeros might be observed among women who had a “low risk” of SA (true zeros) and some among women who had a “high risk” of SA (false zeros), rationalizing the fact that due to fetal chromosome abnormalities, uterine abnormalities, and mutant genes, women do not possess an equal risk of SA. With this more natural consideration, the ZINB model is employed. The better fit of the ZINB over the ZHNB, NB, and PR models suggests that over-dispersion is due to unobserved heterogeneity among women regarding the risk of SA and inflation of zero SA count as well.

The model characteristics are given in Table 1*.* The minimum AIC has been obtained first for the ZINB model, and then for the ZHNB model. The other comparative indicators of the model (minimum MSPE, MAPE, and maximum log likelihood) supports the ZINB model over all the other count models. Figure 1 shows the plot of observed minus predicted probability of SA at each count. The PR model underestimated the probability of occurrence of 1 and 9 SA and overestimated the occurrence of 2–7 SA. Figure 1 exhibits that the NB model predicted the number of SA better than the PR model. The lines of difference between observed minus predicted probability of SA for ZHNB and ZINB were close to the reference zero line, reflecting a better fit of the ZHNB and ZINB models than the other count models. However, from Fig. 1*,* we can deduce that the ZINB model is the best fit out of all the models to predict the number of SA. Figure 2 exhibits the prediction errors for ZINB model among various states (Delhi, Haryana, and Uttar Pradesh) and shows that the model (ZINB) fits the data well for all these states with least prediction error for the state of Harayana in India.

Table 3 exhibits the estimates of regression coefficients corresponding to various cofactors for different count models. Taking the above mentioned model comparison into account, we need to focus on the interpretation offered by ZINB model since it provides the best fit for predicting the number of SA compared to the other models.

**Table 3:** Estimates of regression coefficients corresponding to various cofactors for different count models

Models→	General Linear Models		Zero Hurdle NB Model		Zero Inflated NB model	
Variables↓	Poisson Model	Neg. Binomial Model	Truncated NB Portion (Risk ratio)	Hurdle Portion (Odds Ratio)	Count Portion (Risk ratio)	Zero inflation Portion (Odds Ratio)^a^
**ANC Place**						
Government	Ref.	Ref.	Ref.	Ref.	Ref.	Ref.
Private	1.18 (.083)	1.17 (.070)	1.09 (.120)	1.01 (.231)	1.08 (.086)	1.00 (.172)
Others	1.41*(.067)	1.47 **(.057)	1.56***(.097)	1.50*(.179)	1.59***(.069)	0.47*(.146)
**Place of Residence**						
Rural	Ref.	Ref.	Ref.	Ref.	Ref.	Ref.
Urban	0.91 (.041)	0.91* (.033)	0.97* (.060)	0.94* (.182)	0.87** (.041)	1.12* (.082)
**Age**	1.01 (.002)	1.01 (.002)	1.04 (.004)	1.01 (.028)	1.03 (.002)	0.95 (.012)
**Age at Marriage**	0.98***(.007)	0.98***(.005)	0.96***(.011)	0.95* (.034)	0.98** (.007)	1.04* (.011)
**Total Children**	1.24 (.021)	1.38** (.017)	1.17***(.034)	1.19** (.074)	1.16***(.022)	0.92** (.046)
**Education**						
Illiterate	Ref.	Ref.	Ref.	Ref.	Ref.	Ref.
Primary	1.02 (.049)	1.04 (.040)	0.89* (.079)	0.84 (.190)	0.99* (.050)	1.01 (.105)
Secondary	1.02 (.054)	0.98 (.043)	0.89 (.084)	0.81 (.210)	0.91* (.054)	1.03 (.115)
Higher	0.90 (.079)	0.90*(.063)	0.72***(.115)	0.58**(.410)	0.89***(.078)	1.20**(.160)
**Religion**						
Sikh	Ref.	Ref.	Ref.	Ref.	Ref.	Ref.
Others	0.97 (.040)	0.95 (.032)	0.91 (.052)	0.92 (.174)	0.90* (.040)	1.15* (.088)
**Caste**						
SC/ST	Ref.	Ref.	Ref.	Ref.	Ref.	Ref.
OBC	1.02 (.057)	1.02 (.045)	1.08 (.089)	1.11 (.227)	1.01 (.061)	0.98 (.114)
General	1.02 (.042)	1.01 (.034)	1.08 (.065)	1.09 (.164)	1.01 (.042)	0.99 (.081)
**Wealth Index**						
Low	Ref.	Ref.	Ref.	Ref.	Ref.	Ref.
Middle	0.94 (.067)	0.96 (.054)	0.91* (.098)	0.89* (.176)	0.90* (.071)	0.97* (.137)
High	1.01 (.071)	1.10*(.056)	1.12* (.066)	1.09**(.061)	1.08***(.049)	0.89***(.050)
**Media Exposure**						
Yes	Ref.	Ref.	Ref.	Ref.	Ref.	Ref.
No	1.05* (.078)	1.05 (.064)	1.11 (.116)	0.95 (.265)	1.08 (.089)	0.97 (.163)

^**a**^ The odds ratio for number of SA in low risk group, Value in bracket () gives the S.E

* Significant at 10% level (*p*-value < 0.10)

** Significant at 5% level (*p*-value < 0.05)

***Significant at 1% level (*p*-value < 0.01)

In the ZINB model, the results of both parts of the model together help in understanding the role of the factors on SA count distribution. The zero-inflation portion refers to the logistic part showing the probability of zero SA (not having SA) corresponding to the co-factors, given that the women are in a low-risk group. Therefore, the interpretation of the coefficients in ZINB quite differs from that of ZHNB model. For example, the women who receive ANC from the government institution may have 53% more chance of having zero SA or not having a Spontaneous abortion than the women who had not received ANC from any institution (Government/Private). In the same manner, it is observed that dwelling in urban areas, the late marriage of women, having fewer children, higher education of women, and better wealth condition significantly increase the chances of not facing SA among the women.

The NB portion (Count Portion) of the ZINB model provides the risk of a greater number of SA, given that the women are in a high-risk group. It is observed that women who do not receive ANC from any institution (Government/Private) may have a 59% higher risk of more SA than those who receive ANC from a government institution. Women dwelling in urban settings have 13% less risk of higher number of SA compared to women dwelling in rural areas. Each year increment in the women’s age at marriage may reduce the chance of a higher number of SA by 2%. Results show that each higher order birth to the women may cause to 16% higher likelihood of having a larger number of SA [21] derived various contraceptive policies which ma y be adopted by the policymakers in the region in order to control the population growth. As compared to women without education, women possessing primary, secondary, and higher education have 1, 9, and 11% less chance respectively of having a higher number of SA. Women from the Sikh religion are 10% more likely to have a higher number of SA as compared to the women belonging to other religions. The chances of increased SA are 10% lower among women belonging to the middle wealth status and 8% higher among women belonging to the high wealth status families, compared to the low wealth index women.

The ZINB model has been validated with the latest data of NFHS-4, 2015–16 for a few northern states like Delhi, Haryana, and Uttar Pradesh. It has been observed that the ZINB model fits well for all the mentioned Indian states. For Haryana, it gives the least prediction error. The cultural similarity between the state of Punjab and Haryana can be the rationale behind such findings.

## Discussion

Miscarriage is one of the most critical and prognostic factors for women’s reproductive complications. We need to predict the number of SA among the women of Punjab, Delhi, Uttar Pradesh, and Haryana to improve their reproductive health. As per the available literature, very few studies have been conducted to predict the number of SA among women for different populations using various statistical models. As far as count data is concerned, studies have been undertaken to find excess variability in the distribution of the outcome count variable than what is expected with a Poisson model, and have therefore used the NB model to fit and predict the count data [22, 23]. However, data involving the number of SA often contain surplus zeros, which cause over-dispersion in the data. There is, therefore, a need to inspect fitting zero-hurdle and zero-inflated models, which can also assess the variability due to surplus zeros.

In this study, we have fitted several count models to investigate the causes of over-dispersion and to assess the predictive power of these models concerning the count of SA among women in the Punjab state of India. We have also explained the significance of using zero-inflated models in predicting the number of SA data involving both kinds of zeros. The ZINB count regression models have been found to provide the best fit for the number of SA predictions among the women of Punjab and other northern states in India. Thus, the data of the number of SA possess over-dispersion not only due to surplus zeros but also due to unobserved heterogeneity among women in the region. The Poisson Regression model has been found to have the highest prediction error for predicting SA frequency due to the presence of over-dispersion. On the other end, the ZINB/ZHNB models assume more than one source of over-dispersion and have provided a smaller prediction error.

The ZINB and ZHNB models are similar in identifying factors associated with the number of SA as well as in predicting the count of the event. In the current paper, we have focused on predicting the number of SA. As such, either model could have been used to predict the number of SA. The ZHNB model may be preferred over ZINB since it is easier to interpret the results; however, for the phenomenon under study, ZINB is preferred because of having less prediction error. The above findings are supported by [24], who has also found the harmony between these two models on the data of vaccine. The study suggests that model selection should follow the study objectives and the structure of the target variable. [25], however, has recommended that models should be selected depending on the logic behind the data generating mechanism.

A few studies have observed that the distribution of the count variable often contains some proportion of sampling/false zeros, which may arise in the high-risk group [26, 27]. Considering the heterogeneity among women, it makes sense to consider zeros to be a mixture of structural zeros and sampling zeros, which allows the use of the zero-inflated models. The ZINB model estimates the sampling zero SA among the women who are at high risk of SA and also does a slightly better job than the ZHNB model when it comes to predictive performance.

The MSPE is 68.74 and 6.89% less, using the ZINB model in comparison to the NB regression model and the ZHNB model, respectively. Additionally, the prediction accuracy of the ZINB model is remarkably better than that of the NB model, stipulating that the Negative Binomial model may not be acceptable for describing the distribution of SA in future. In a nutshell, we may conclude that ZINB count regression model is the best choice for the policymakers to predict the incidence of SA in above mentioned northern states of India.

It is pertinent to create a sound strategy to classify women at high-risk and low-risk of SA so that the women at high risk can get more attention from clinicians and family members. It would be a fruitful but challenging task for clinical practitioners and medical scientists to observe the heterogeneity of SA risk among women considering their biological parameters. However, these diagnoses may involve a massive amount of money, which may be difficult to bear for the general population of the Indian states. Therefore, there should be a government policy to provide these kinds of diagnoses at a reasonable cost; this is a matter of future research as well.

Taking into account that the data used for the model building is a cross-sectional survey, it is expected to provide the association not a causality, that prevents the interpretation concerning a causal relationship. Besides the determinants used in the study, many other factors may be associated with SA. Thus, a further investigation can be done to improve the suggested model.

## Conclusions

This study is a rare analysis to observe patterns of the number of SA among women of Punjab and a few northern states, using such a large dataset collected in India (DLHS-4, 2012–13 & NFHS-4, 2015–16). In our study, woman’s education, total children born to a woman, ANC place, place of residence, and economic status among women are associated with a high risk of SA and with a higher number of SA. Along with these factors, religion has also been found to be associated with an increased risk of a higher number of SA, given that the women are at high-risk of SA.

As a concluding note, we can state that ZINB count regression model can be utilized to predict the number of SA more accurately than other count regression models. The ZINB model more accurately estimates the sampling zeros and has a comparatively lower prediction error as compared to the rest of the models. Thus, the ZINB model may be considered to be the best model for predicting and describing the number of SA. Successful validation of the ZINB model for other northern states suggests the scope to generalize the model for the Indian scenario. The study advocates promoting higher education among women in Punjab and other northern states in India; it also suggests women receiving institutional ANC, and attain lower parity to have better reproductive health. In this connection, policymakers should cascade the knowledge about contraceptives among couples in northern India, especially in the regions where the education status is meager.

## Data Availability

The data is available on request from the DHS website.

## Abbreviations

SA: Spontaneous abortion
DLHS: District level household and facility survey
NFHS: National family health survey
PR: Poisson regression
NB: Negative binomial
ZHNB: Zero hurdle negative binomial
ZINB: Zero-inflated negative binomial
ANC: Antenatal care
AIC: Akaike information criterion
MSPE: Mean squared prediction error
MAPE: Mean absolute prediction error
